# Urinary Tract Infection Causing Bradycardia, Renal Failure, Atrioventricular Nodal Blockade, Shock, and Hyperkalemia (BRASH) Syndrome: A Case Report and a Brief Review of the Literature

**DOI:** 10.7759/cureus.27641

**Published:** 2022-08-03

**Authors:** Ramakanth Pata, Innocent Lutaya, Molly Mefford, Amita Arora, Nway Nway

**Affiliations:** 1 Pulmonary and Critical Care Medicine, One Brooklyn Health, New York, USA; 2 Pulmonary and Critical Care Medicine, University of Cincinnati Medical Center, Cincinatti, USA; 3 Medicine, American University of Antigua, New York, USA; 4 Medicine, Interfaith Medical Center, New York, USA; 5 Internal Medicine, Interfaith Medical Center, New York, USA

**Keywords:** acute kidney injury, acute kidney injury and brash syndrome, av block, uti, brash, hyperkalaemia, shock, av blockade, renal failure, bradycardia

## Abstract

Bradycardia, renal failure, atrioventricular (AV) nodal blockade, shock, and hyperkalemia (BRASH) syndrome commonly occurs in the elderly population with compromised renal function and a history of taking AV nodal blocking agents on a regular basis. Hypovolemia and worsening of renal function are considered to be the major risk factors. BRASH syndrome should be differentiated from pure intoxication with AV nodal blocking agents, as the therapeutic goals of these conditions are different from each other. It encompasses a vicious cycle of bradycardia and decreased cardiac output leading to organ dysfunction including renal failure with hyperkalemia, further augmenting bradycardia. It is usually associated with high morbidity and mortality. Typically, the treatment involves increasing renal blood flow by augmenting cardiac output using catecholamine infusion. Very rarely, interventions such as intralipid emulsion and continuous renal replacement therapy (CRRT) may be required on a case-to-case basis. Promptly recognizing the symptoms of BRASH syndrome can help to avoid diagnostic delays and reduce mortality rates.

## Introduction

BRASH syndrome is a constellation of clinical entities that includes bradycardia, renal failure, atrioventricular (AV) nodal blockade, shock, and hyperkalemia. It usually originates from bradycardia leading to decreased cardiac output and renal failure. Hyperkalemia caused by renal failure is augmented by beta-blockers (especially non-specific beta blockers). This leads to AV blockade and further worsening of renal failure, and thus the synergistic action of hyperkalemia and bradycardia, leading to self-perpetuating AV nodal blockade and renal failure [1]. This syndromic constellation was recently established as a distinctive entity in 2016; however, it is believed that the occurrence of each component has been common since the use of beta-blockers and calcium channel blockers became widespread in the 60s [2]. In this report, we present a case of BRASH syndrome secondary to urinary tract infection (UTI) and its management including high-dose euglycemic insulin therapy and intralipid infusion. A brief review of the relevant literature is also presented.

An abstract of this article has been submitted to be presented at the upcoming 2022 American Heart Association Conference.

## Case presentation

**Figure 1:**
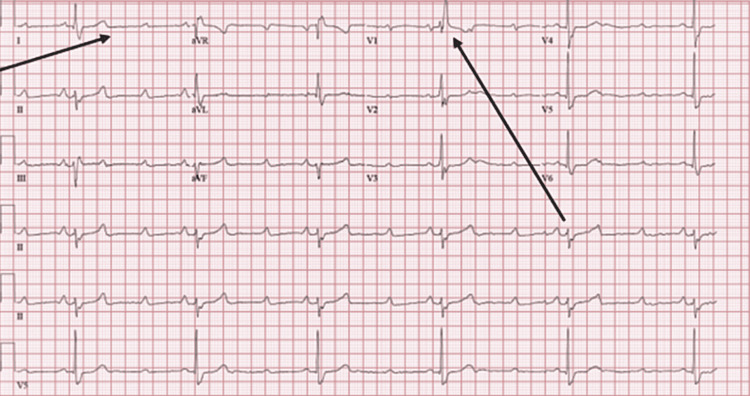
ECG demonstrating bradycardia with a high-grade AV block and bifascicular block ECG: electrocardiogram; AV: atrioventricular

A 58-year-old man was brought to the ED with complaints of altered mental status after he had experienced a fall. His past medical history was significant for coronary artery disease with multiple stents, congestive heart failure with a reduced ejection fraction of 30%, and a history of monomorphic ventricular tachycardia with a single right ventricular lead pacer and implantable cardioverter defibrillator (ICD). His home medications included furosemide 20 mg, spironolactone 25 mg, lisinopril 20 mg, carvedilol 25 mg twice daily for heart failure, and sotalol 80 mg for atrial fibrillation. On arrival, the patient was hypothermic with a temperature of 32 °C, hypotensive with a blood pressure of 70/40 mmHg, and bradycardic with a heart rate of around 30 beats per minute (BPM). A CT scan of the head was negative for any acute intracranial process. An ECG showed a high-grade AV block with a bifascicular block (Figure 1). He had an elevated white cell count of 14.9, with hyperkalemia and acute kidney injury (Table 1). Hyperkalemia and hypotension were managed in the ED with intravenous fluid, calcium, insulin, and dextrose. BRASH syndrome was promptly recognized because the heart rate was still 30 BPM and was unresponsive to atropine. Dopamine infusion was started with a slight improvement in the heart rate to 50 BPM but hypotension and hyperkalemia persisted. The cardiology team was consulted. We believe that a possible cause of the pacer’s inability to correct the bradycardia was the presence of severe electrolyte abnormality, which could have hindered the heart from capturing the pacer. After prompt recognition of the patient's BRASH syndrome, cardiology recommended that he be quickly upgraded to cardiac-centered ICU care.

**Table 1:** Admission laboratory values showing hyperkalemia and acute kidney injury BUN: blood urea nitrogen; EGFR: estimated glomerular filtration rate N: normal; H: high; L: low

Potassium (normal range: 3.5-5.0 mEq/L)	BUN (normal range: 6-20 mg/dl)	Creatinine (normal range: 0.6-1.2 mg/dL)	EGFR (normal level: >60 mL/min/1.73 m^2^)	Anion gap (normal range: 4-12 mEq/L)	CO_2_ (normal range: 23-29 mEq/L)
6.5 (H)	63 (H)	2.15 (H)	25 (L)	8 (N)	22 (N)
6.5 (H)	67 (H)	2.37 (H)	22 (L)	7 (N)	25 (N)
5.7 (H)	73 (H)	2.77 (H)	18 (L)	5 (N)	28 (N)
5.9 (H)	70 (H)	2.67 (H)	19 (L)	9 (N)	24 (N)

Epinephrine, and subsequently norepinephrine and vasopressin (at a fixed dose of 0.04u) were added to achieve the target mean arterial pressure of 65 mmHg. Continuous renal replacement therapy (CRRT) was instituted for renal failure and hyperkalemia. Bradycardia and hypotension persisted, despite maximal doses of catecholamines. As there was a concern regarding beta-blocker intoxication, medical reconciliation was done, which revealed consistent and compliant intake of medications as prescribed. The patient was initiated on 20% intralipid infusion at 0.5 ml/minute after a bolus along with high-dose euglycemic insulin therapy. The high-dose euglycemic insulin therapy was started at 0.5 IU/kg/hr and titrated to a heart rate up to a maximum of 5 IU/kg/hr. There were modest improvements in cardiac output and hemodynamics, monitored by FloTrac®/Vigileo. Further workup then revealed evidence of UTI on urine analysis. Empiric broad-spectrum antibiotics, vancomycin, and piperacillin-tazobactam were started while waiting for culture results.

Over the course of the next 72 hours, the heart rate and hypotension gradually improved. All the infusions were weaned off eventually without the need for transvenous pacing or plasmapheresis. An echocardiogram showed an ejection fraction of 45-50% with mildly overloaded right ventricle and regional wall motion abnormalities. Blood cultures turned out to be negative, but urine cultures returned positive for pan-sensitive E. coli. Vancomycin and piperacillin-tazobactam were discontinued and intravenous ceftriaxone was initiated.

On day four, the patient developed three episodes of atrial fibrillation with rapid ventricular rate and hypotension that transiently terminated with cardioversion. Because of the recurrence, amiodarone and heparin infusion were started. Electrophysiology was consulted, and the patient later underwent ablation for atrial fibrillation. He was eventually discharged on apixaban 5 mg, aspirin 81 mg, and metoprolol 25 mg, to be followed up in the cardiology clinic.

## Discussion

BRASH syndrome represents a vicious cycle involving bradycardia, renal failure, AV nodal block, shock, and hyperkalemia. BRASH syndrome is most commonly initiated by hypovolemia or worsening renal function in a patient consistently taking AV nodal blocking agents [1,2,3,4,5]. BRASH syndrome was recognized as a distinct entity in 2016; however, it is largely underrecognized, with a total of 23 cases reported so far based on a literature review [6,7,8]. The true prevalence of BRASH syndrome is unknown. Although the etiology and epidemiology of this condition are not well defined, older age, poor kidney function, and the use of AV nodal blocker medications were the common findings in a clinical review of 23 cases and may therefore be considered contributing factors [8,9]. A recent review of 27 case reports indicates that BRASH syndrome is found predominantly in elderly patients (average age of 68 years) with all 32 patients having underlying cardiac comorbidities including atrial fibrillation and heart failure [10]. Furthermore, patients taking multiple AV nodal blocking medications, as well as angiotensin-converting enzyme (ACE) inhibitors or angiotensin II receptor blockers (ARBs) have an increased risk of developing BRASH syndrome [11]. We presented a case of BRASH syndrome likely caused by the accumulation of AV nodal blocking agents with superimposed acute kidney injury as a result of UTI. BRASH syndrome should be differentiated from intoxication with AV nodal blocking agents because the management and therapeutic goals are different.

Although the exact pathophysiology of BRASH syndrome is unclear, it is thought to involve synergistic effects between hyperkalemia and AV nodal block secondary to underlying renal failure, resulting in bradycardia and hypotension [1,5,7]. Bradycardia causes reduced cardiac output with poor renal perfusion, which further worsens renal failure and thus hyperkalemia, resulting in a vicious cycle [2,5,12]. Hyperkalemia is further augmented by beta-blocking effects. Of note, 80% of patients with BRASH syndrome initially present with syncope, dizziness, and generalized weakness [11]. It is most often seen in elderly patients with multiple underlying comorbidities, such as cardiac disease, chronic kidney disease, and hypertension [6]. Our patient reported a history of progressive generalized weakness for two weeks prior to the presentation.

The clinical presentation for BRASH syndrome is variable, ranging from asymptomatic bradycardia to multi-system organ failure requiring extracorporeal therapies in rare cases [7,8], and thus poses a diagnostic challenge [12-14]. In most cases, patients present with minimal symptoms initially and appear well, despite low blood pressure and low heart rate [1,2].

The complete metabolic panel and ECG assessment are the integral investigations used in the evaluation of patients with BRASH syndrome [4,9]. In addition, a bedside limited ultrasound is crucial in determining the volume status of these patients [1,12,15]. BRASH syndrome must be differentiated from an overdose of AV nodal blocking agents and isolated hyperkalemia There may be an overlap in many features including treatment of intoxication of AV nodal blocking agents, BRASH syndrome, and hyperkalemia. However, it is essential to differentiate, as the therapeutic priorities of each condition are often different from each other (Table 2).

**Table 2:** Comparison of clinical features and management of AV nodal blocking overdose, BRASH syndrome, and hyperkalemia BRASH: bradycardia, renal failure, atrioventricular nodal blockade, shock, and hyperkalemia; ECG: electrocardiogram; AV: atrioventricular; ACE: angiotensin-converting enzyme

Salient features	Beta-blocker intoxication	BRASH syndrome	Hyperkalemia
History and presentation	History of accidental or intentional ingestion of AV nodal blocking agents	Hypovolemia or worsening renal dysfunction is usually a precipitant. Hx of good adherence to prescribed medications	Non-compliance to medications or dialysis therapy
Comorbidities	No or mild baseline renal dysfunction	Pre-existing baseline renal dysfunction and cardiac comorbidity	End-stage renal disease
ECG findings	Severe bradycardia and heart block are usually seen. Hyperkalemia may or may not be present along with ECG changes	Severe bradycardia or junctional rhythm unexplained by the degree of hyperkalemia. QRS widening and peaked T waves less prominent	Hyperkalemia is prominent. Bradycardia can be seen with severe hyperkalemia but is almost always accompanied by other ECG changes of hyperkalemia (QRS widening, peaked T waves)
Management	Management is predominantly focused on supporting the hemodynamics until the medications are cleared. Includes glucagon, catecholamine infusion, euglycemic high-dose insulin therapy, and intralipid therapy	Management is predominantly focused on improving renal blood flow. Hyperkalemia: IV calcium, insulin/dextrose, albuterol IVF or diuretics based on volume status. Fludrocortisone if on ACE inhibitors. If hypotension: epinephrine; normal BP: isoprenaline	Management is focused on removing potassium. Intravenous calcium, insulin/dextrose, albuterol nebs, emergent dialysis or diuretics, or potassium chelators depending on the severity
Extracorporeal therapies	Transvenous pacing or plasmapheresis may be needed	Pacing, plasmapheresis, intralipid, and high-dose insulin infusion are required in rare cases	Emergent renal replacement therapy may be required

In isolated hyperkalemia, the associated bradycardia is most often seen with potassium levels above 6.5 mEq/L [3]. In contrast, BRASH syndrome patients often present with profound bradycardia at only mild-moderate hyperkalemia [5,8]. Classic ECG findings at such high levels of potassium include peaked T-waves, flat P-waves, PR prolongation, and/or QRS widening [9]. However, BRASH syndrome patients typically show profound bradycardia without the classic ECG features of hyperkalemia. A study showed that verapamil may induce junctional bradycardia in the presence of even mild hyperkalemia [5]. Likewise, another study described a patient on diltiazem who developed BRASH syndrome in the presence of mild hyperkalemia [3]. Of note, patients with BRASH syndrome and intoxication with AV nodal blocking agents have paradoxically only modest elevation of lactate out of proportion to the degree of hypotension although acidosis may be severe. This paradoxically low lactate is thought to be secondary to the effect of beta-blockers on glycolysis. Acidosis could be secondary to renal dysfunction.

The main goal for the treatment of BRASH syndrome is to increase renal blood flow by augmenting cardiac output [13]. Acute mild cases of BRASH syndrome can be managed with non-invasive interventions, including treatment of hyperkalemia, management of bradycardia, and volume resuscitation [13,14,16,17]. Initial management is focused on the life-threatening components, with interventions including the management of bradycardia and hyperkalemia [1,11]. Management of hyperkalemia involves IV calcium, insulin + D50, and beta-agonists [13,17,18]. By administering IV calcium, stabilization of the cardiac membrane is achieved, thus correcting the bradycardia [8,10]. However, if this fails to correct the bradycardia, an epinephrine infusion is recommended [8]. Volume status determines whether the patient would benefit from either aggressive diuresis or crystalloid resuscitation. Bedside echocardiography may be crucial in determining the volume status along with other dynamic measures of preload responsiveness. Furosemide is the diuretic of choice and fludrocortisone 0.2 mg may be added if there is a history of ACE inhibitor/ARB intake. Most patients who take ACE inhibitors/ARBs may have low aldosterone levels that may benefit from the administration of fludrocortisone to excrete potassium. Fluid resuscitation can be done with crystalloid administration depending on a strong ion gap (SIG). SIG is typically calculated by subtracting strong cations from strong anions. The choice of crystalloids includes normal saline, plasmalyte, ringers lactate, and isotonic bicarbonate. pH-guided volume resuscitation has also been described in the literature. For example, in patients with uremic acidosis, isotonic bicarbonate may be considered [8,15]. For patients with severe BRASH syndrome and/or developing complications, the management includes hemodialysis and/or transvenous pacing [18]. 

Catecholamine infusion must be considered to raise mean arterial pressure (monitored by arterial line) and cardiac output. Non-invasive or minimally invasive cardiac output monitors must be considered to titrate catecholamine infusions. Typically, epinephrine or dopamine infusion must be considered for inotropic or chronotropic action and Levophed or vasopressin may be added for vasoconstrictive properties. Typically, in patients taking pure beta-blockers, epinephrine infusion alone may be sufficient. In patients taking combined alpha + beta-blockers such as labetalol or carvedilol, vasopressors may be required in addition to inotropic/chronotropic augmentation. Our patient was on a maximal dose of carvedilol, which is a combined alpha and beta-blocker.

Many patients with BRASH syndrome usually respond to the above therapies. However, occasionally, patients may require some therapies similar to those for beta-blocker overdoses such as intralipid emulsion, euglycemic high-dose insulin therapy, and transvenous pacing. Some extracorporeal therapies such as CRRT and plasmapheresis may also be needed on a case-by-case basis. Our patient remained refractory to catecholamine infusion, requiring intralipid emulsion infusion and euglycemic high-dose insulin therapy. These therapies are associated with triglyceride and electrolyte abnormalities that need constant monitoring and aggressive management.

Collectively, the severity of acute kidney injury and hyperkalemia are the major components of BRASH syndrome, influencing prognosis [1]. Hyperkalemia has been correlated with poor outcomes in numerous settings, including patients with cardiac and renal disease as well as acutely ill patients [10]. Overall, prompt recognition and aggressive medical management greatly improve the prognosis and often eliminate the need for additional invasive interventions. For the majority of cases, the clinical course is uncomplicated with a reversal of symptoms in 24-48 hours after treatment [15,16]. In a recent review, the majority of patients recovered back to baseline; however, seven of the 30 patients further developed end-stage renal disease requiring renal replacement therapy (RRT) or conduction abnormalities requiring lifelong follow-up, and two died from cardiac arrest [17,18].

## Conclusions

Hyperkalemia, bradycardia, and renal failure are frequently encountered in emergency and critical care settings. It is imperative to recognize the diagnosis as BRASH syndrome promptly because it can present as refractory shock with bradycardia, which does not respond to the standard advanced cardiovascular life support (ACLS) algorithm, as was the case in our presentation. By aggressive hemodynamic support and control of hyperkalemia, we can prevent further complications like multiorgan dysfunction or death. One also needs to pay attention to underlying cardiac comorbidities once AV nodal blocking agents are discontinued, as indicated by our case who developed atrial fibrillation.
